# Supplementary material for: Mechanism of REST/NRSF regulation of clustered protocadherin α genes
Source: Nucleic Acids Res. 2021 Apr 13;49(8):4506–21. doi: 10.1093/nar/gkab248 (PMC8096226; doi:10.1093/nar/gkab248)
Supplement: gkab248_Supplemental_Files [file gkab248_supplemental_files.zip › Supplementary Figures.pdf]

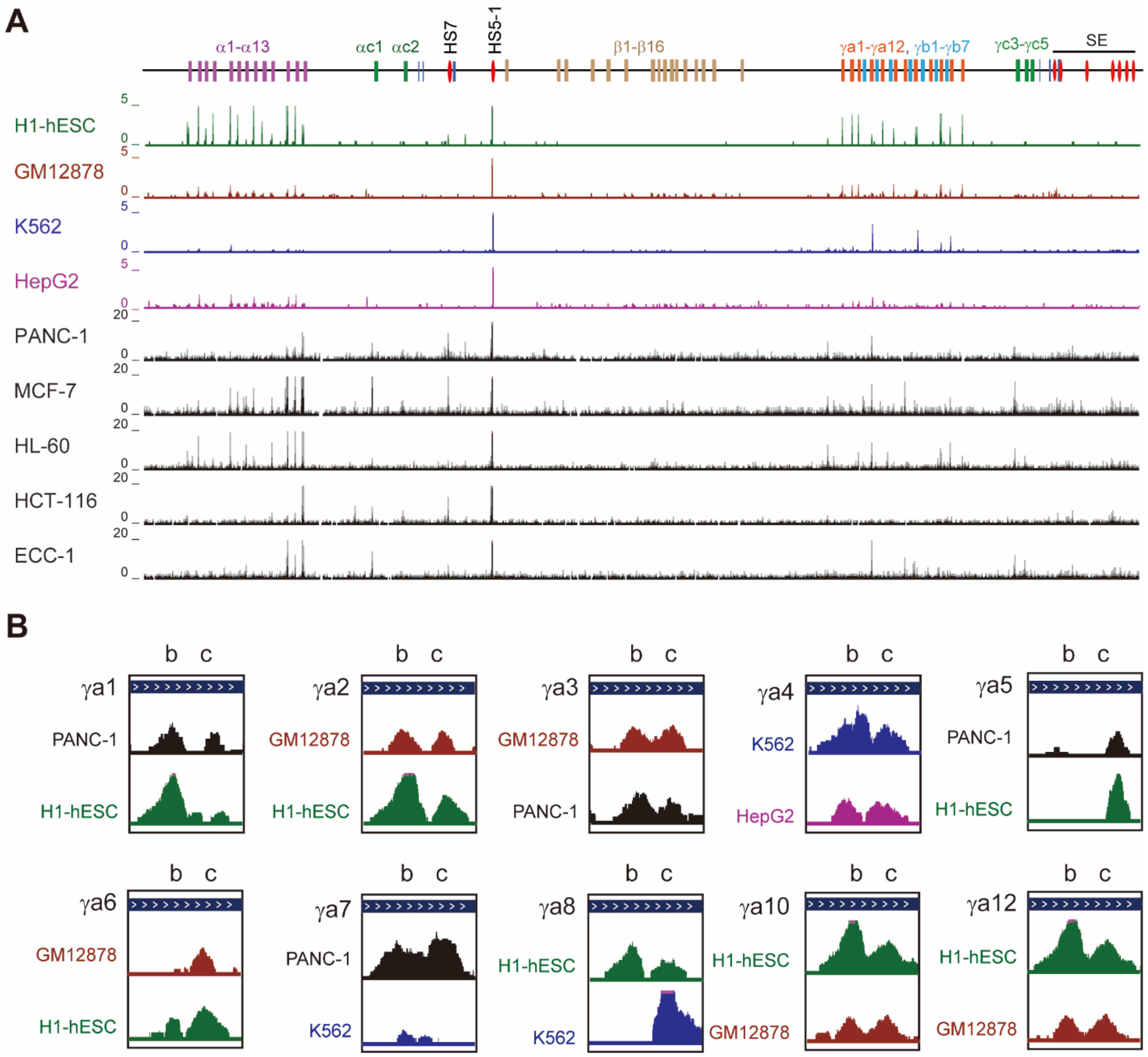

Supplementary Figure S1. REST/NRSF binding patterns in the three human PCDH clusters. (**A**) REST/NRSF binding in clustered *PCDH* in different cell lines (data are from the ENCODE project). (**B**) Showing are the two REST/NRSF peaks in the *PCDHγa* alternate exons.

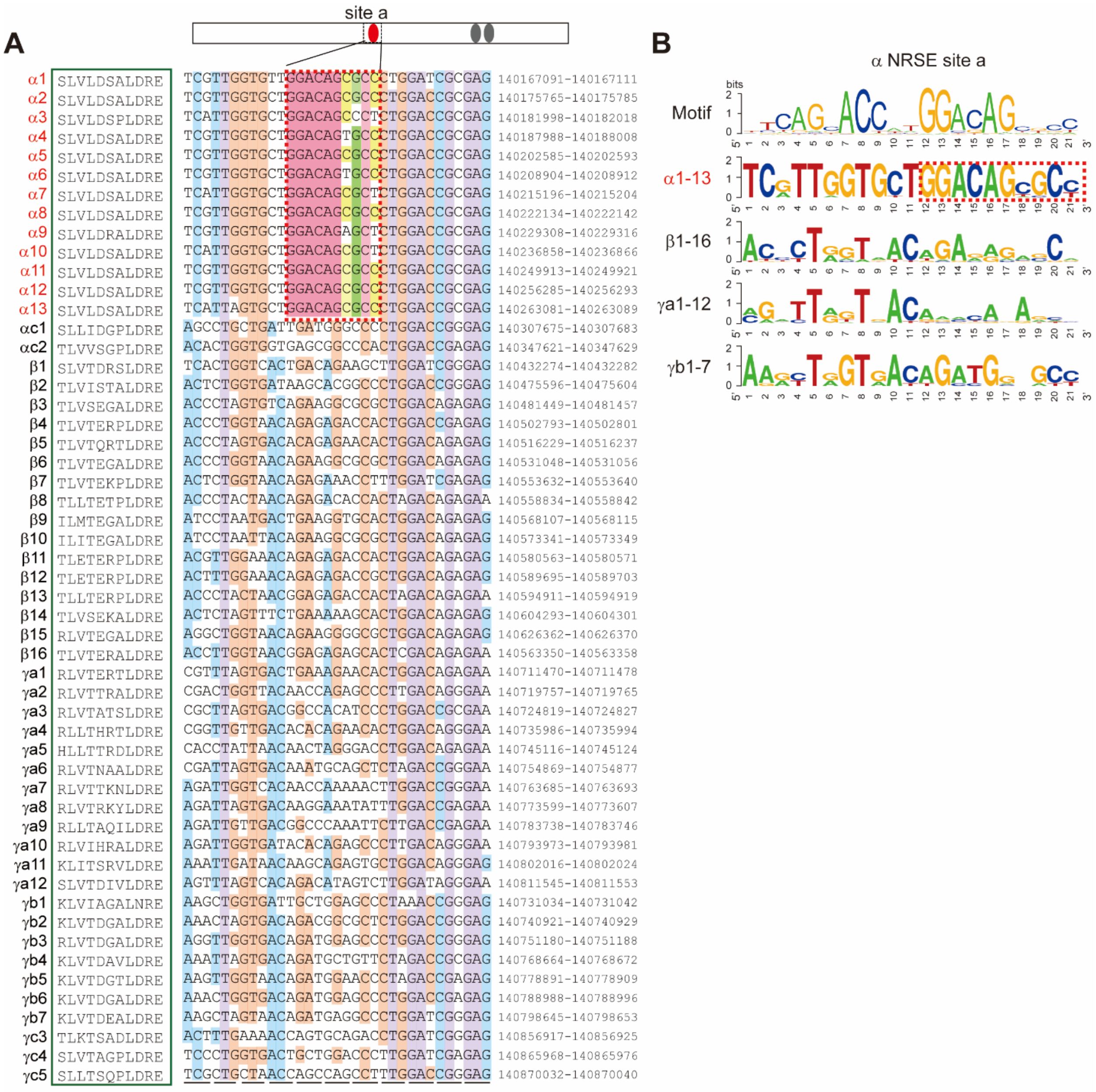

Supplementary Figure S2. Sequence alignment of site 'a' in clustered *PCDH* variable exons. (**A**) Alignment and genomic location of the sequences in the 'a' site of the clustered *PCDH* variable exons. (**B**) Comparison of site 'a' consensus sequences of *PCDH* subclusters. Note that only alternate members of the *PCDHα* cluster contains NRSE site 'a'.

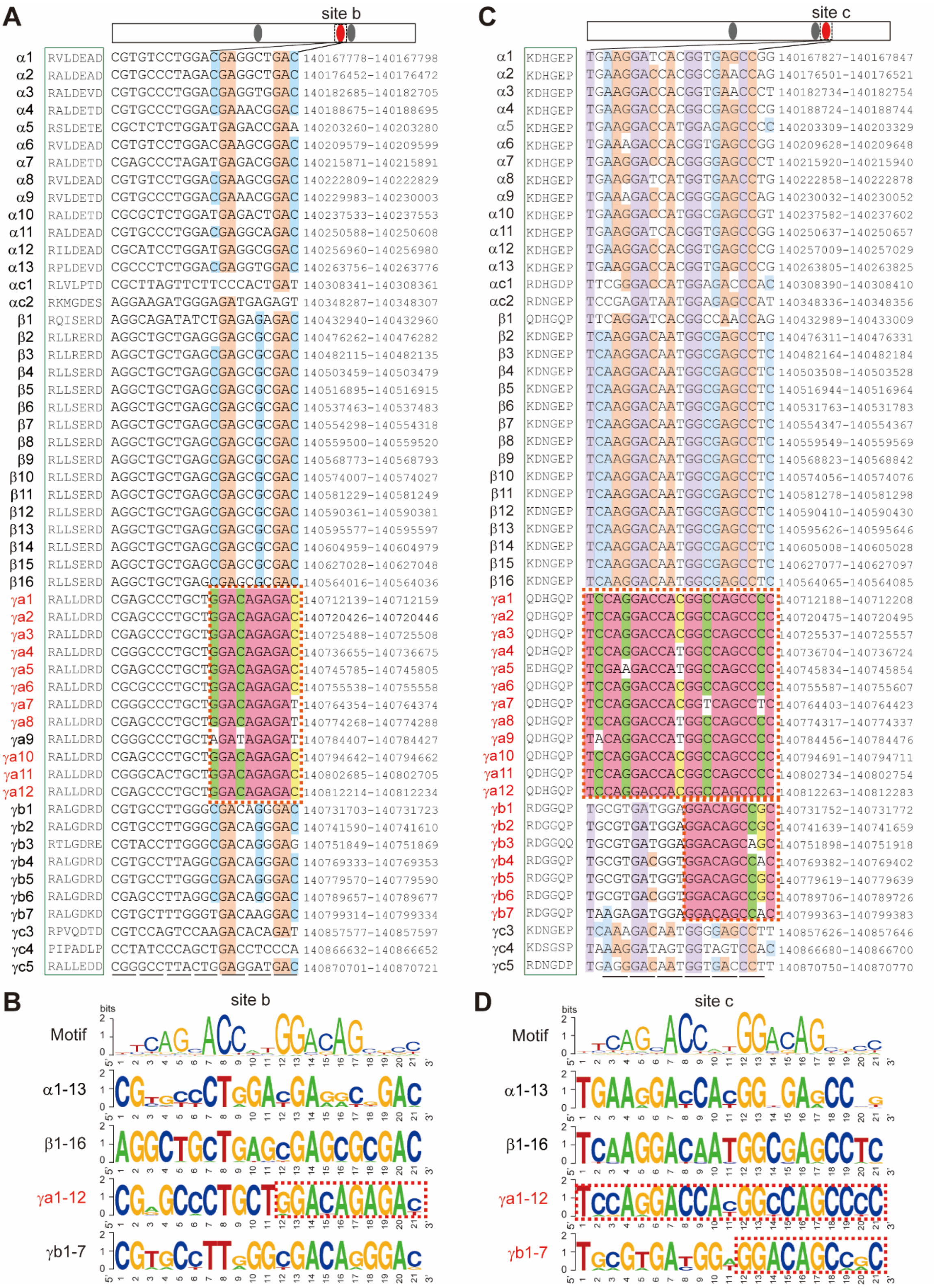

Supplementary Figure S3. Sequence alignment of sites 'b' and 'c' in clustered *PCDH* variable exons. (**A**) Alignment and genomic location of the sequences in the site 'b' of the clustered *PCDH* variable exons. (**B**) Comparison of site 'b' consensus sequences of *PCDH* subclusters. (**C**) Alignment and genomic location of the sequences in the site 'c' of the clustered *PCDH* variable exons. (**D**) Comparison of site 'c' consensus sequences of *PCDH* subclusters.

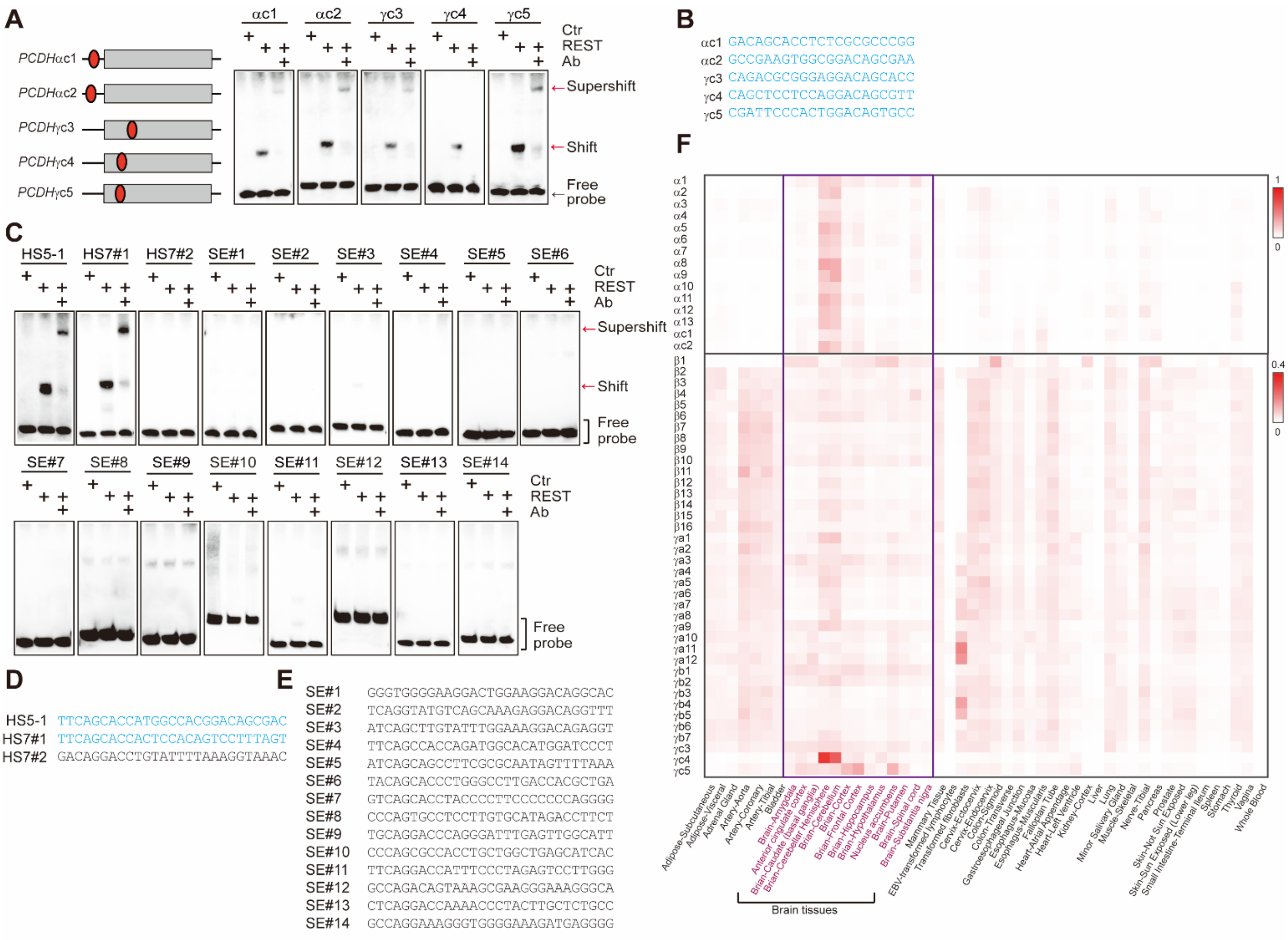

Supplementary Figure S4. REST/NRSF binding profiles in C-type genes and super-enhancers. (**A**) EMSA assays of REST/NRSF with the NRSE probes of C-type *PCDH* genes, using mock protein as a control. (**B**) The sequences of the NRSE motif in the C-type *PCDH* genes. (**C**) EMSA assays of REST/NRSF with putative NRSE probes within the super-enhancers, using mock protein as a control. (**D**) Probe sequences in the super-enhancer of *PCDHα*. (**E**) Probe sequences in the super-enhancer of *PCDHβγ*. (**F**) The expression levels of the clustered *PCDH* genes in different tissues.

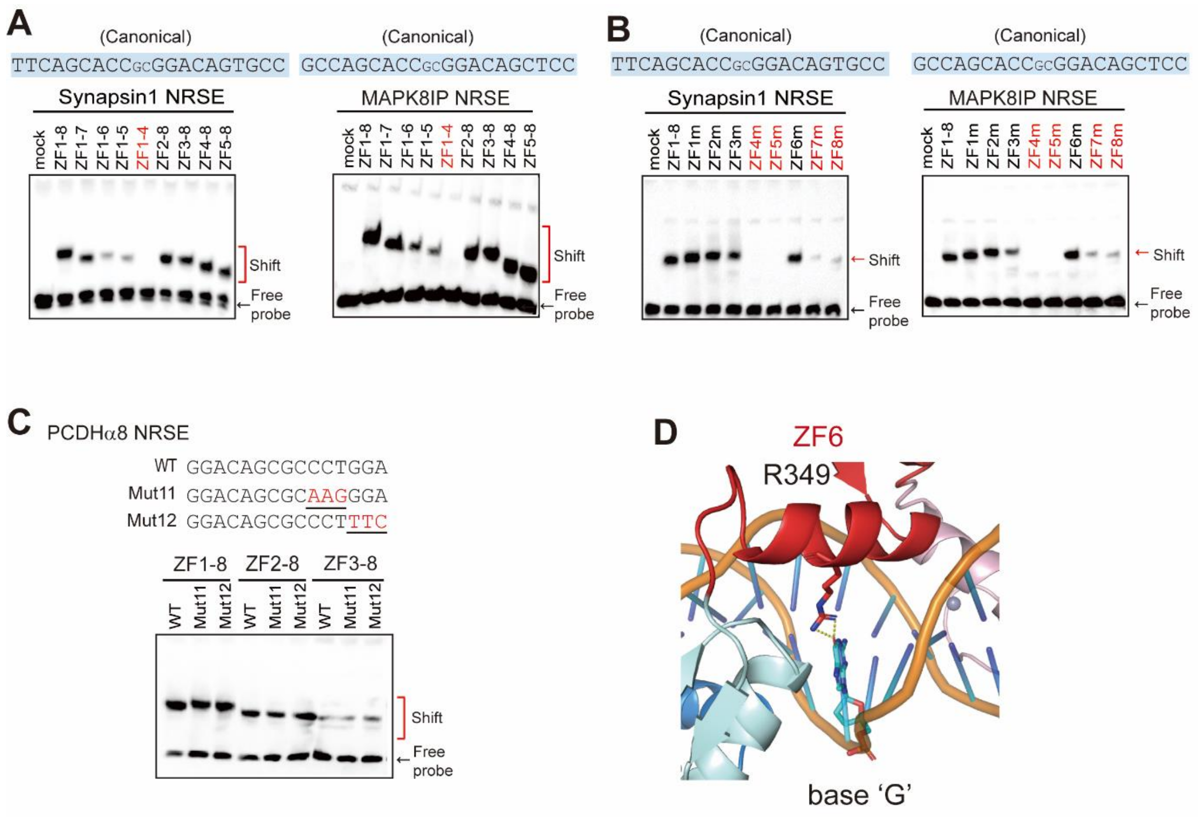

Supplementary Figure S5. Binding patterns of ZF-mutated or ZF-deleted REST/NRSF with different probes. (**A**) EMSA assays for the binding of ZF-deleted REST/NRSF with two canonical NRSEs. (**B**) EMSA assays for the binding of ZF-mutated REST/NRSF with two canonical NRSEs. (**C**) EMSA assays for the ZF-deleted REST/NRSF with the Mut11 and Mut12 probes of *PCDHα8* NRSE. (**D**) Shown are the contacts of R349 in ZF6 with the base 'G' in the complementary strand at the 9th position of the canonical *PCDHγa6* NRSE.

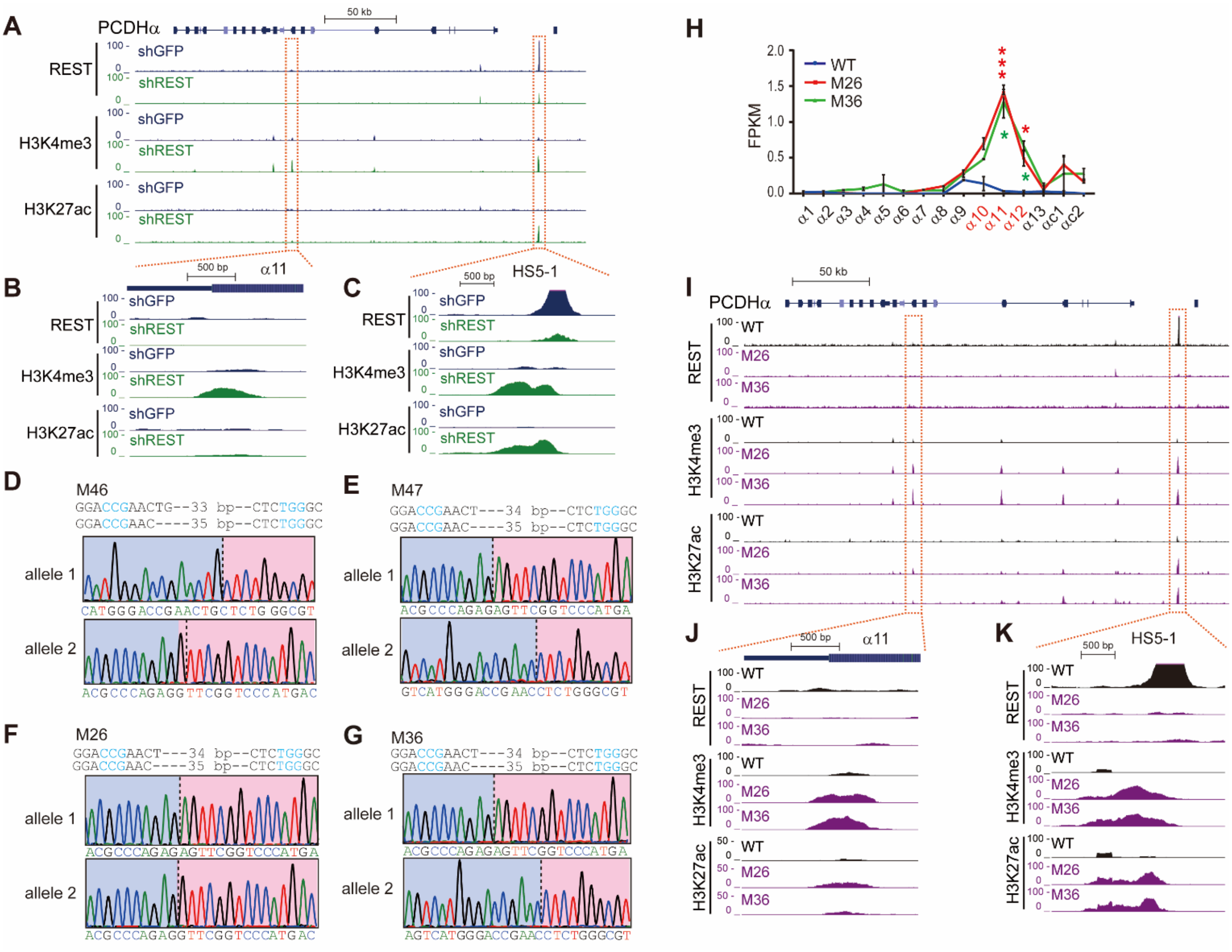

Supplementary Figure S6. Genotyping and CRISPR screening of single-cell NRSE deletion clones by DNA-fragment editing in HEK293T and HEC-1-B cells. (**A**-**C**) ChIP-seq with a specific antibody against REST/NRSF, H3K4me3, or H3K27ac in HEK293T cells upon knockdown of REST/NRSF by shRNA. (**D**, **E**) Genotyping of the *HS5-1* NRSE-deleted single-cell clones of HEC-1-B cells (M46, M47). The PAM sites are highlighted. (**F**, **G**) Genotyping of the *HS5-1* NRSE-deleted single-cell clones of HEK293T cells (M26, M36). The PAM sites are highlighted. (**H**) Expression profiles of *PCDHα* measured by RNA-seq in HEK293T single-cell CRISPR clones. Data are presented as mean ± SEM. * $P < 0.05$, *** $P < 0.001$. (**I**-**K**) ChIP-seq of REST/NRSF, H3K4me3, and H3K27ac in HEK293T single-cell CRISPR clones upon deletion of the *HS5-1* NRSE.

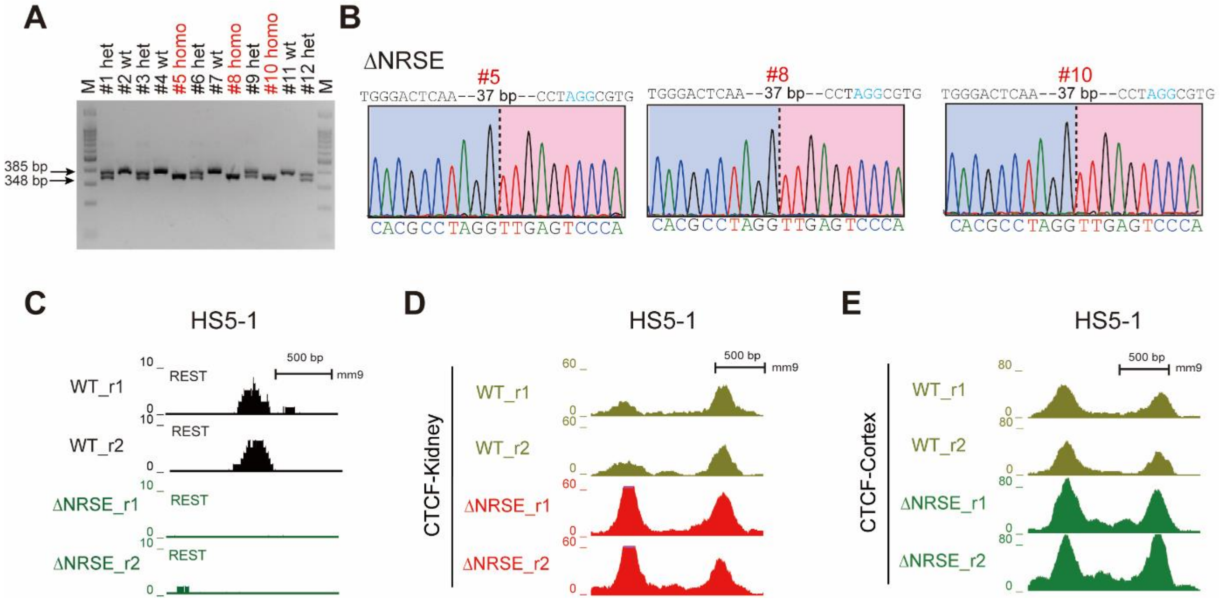

Supplementary Figure S7. Genotyping and CTCF binding of the *HS5-1* NRSE-deleted mice by DNA-fragment editing. (**A**, **B**) Genotyping of P0 mice with the *HS5-1* NRSE deletion. (**C**) ChIP-seq confirmed the abolishment of REST/NRSF binding in the *HS5-1* enhancer upon NRSE deletion in mice. (**D**) CTCF ChIP-seq peak of *HS5-1* in the kidney tissues from WT and *HS5-1* NRSE-deleted mice. (**E**) CTCF ChIP-seq peak of *HS5-1* in the cortical tissues from WT and *HS5-1* NRSE-deleted mice.

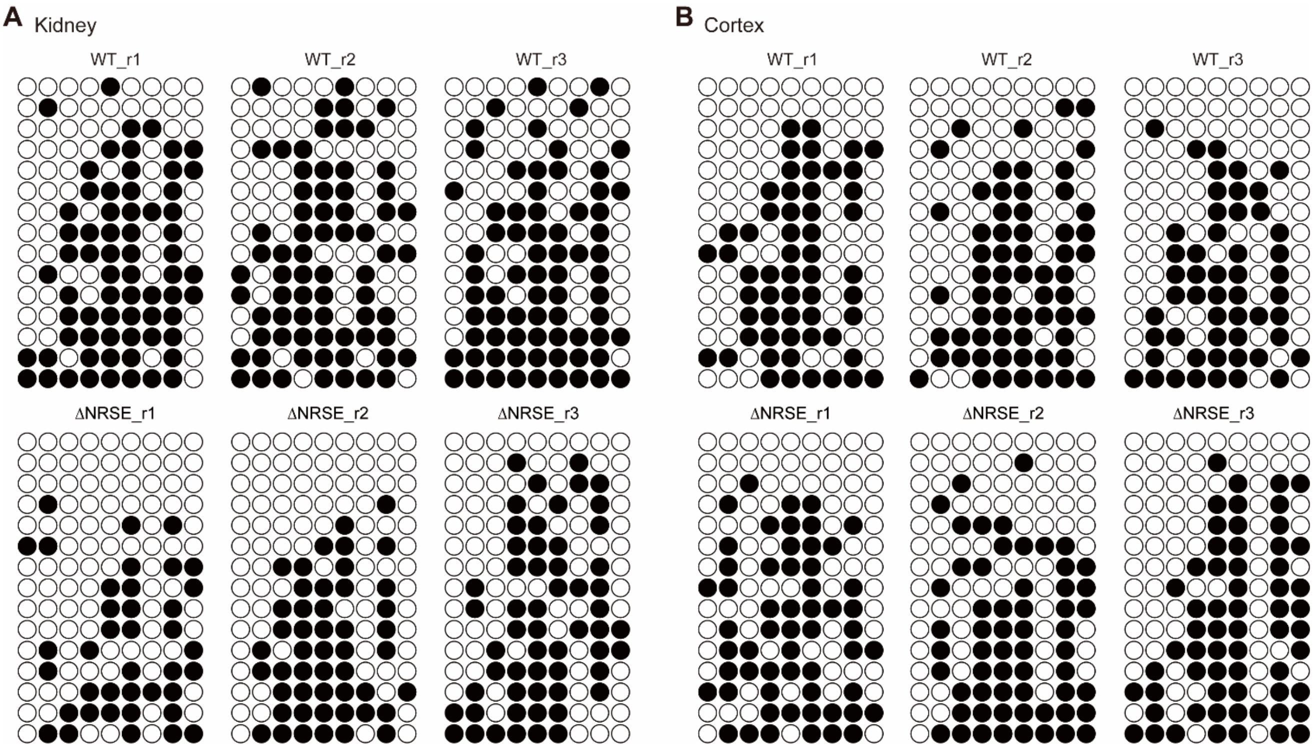

Supplementary Figure S8. DNA methylation analysis of the *Pcdh*α*9* gene by bisulfite sequencing with mouse kidney (**A**) and cortical (**B**) tissues after *HS5-1* NRSE deletion. Both wildtype and mutant mice were sequenced with three replicates. The open circles represent the unmethylated cytosine of CpG, whereas filled circles represent the methylated cytosine of CpG.

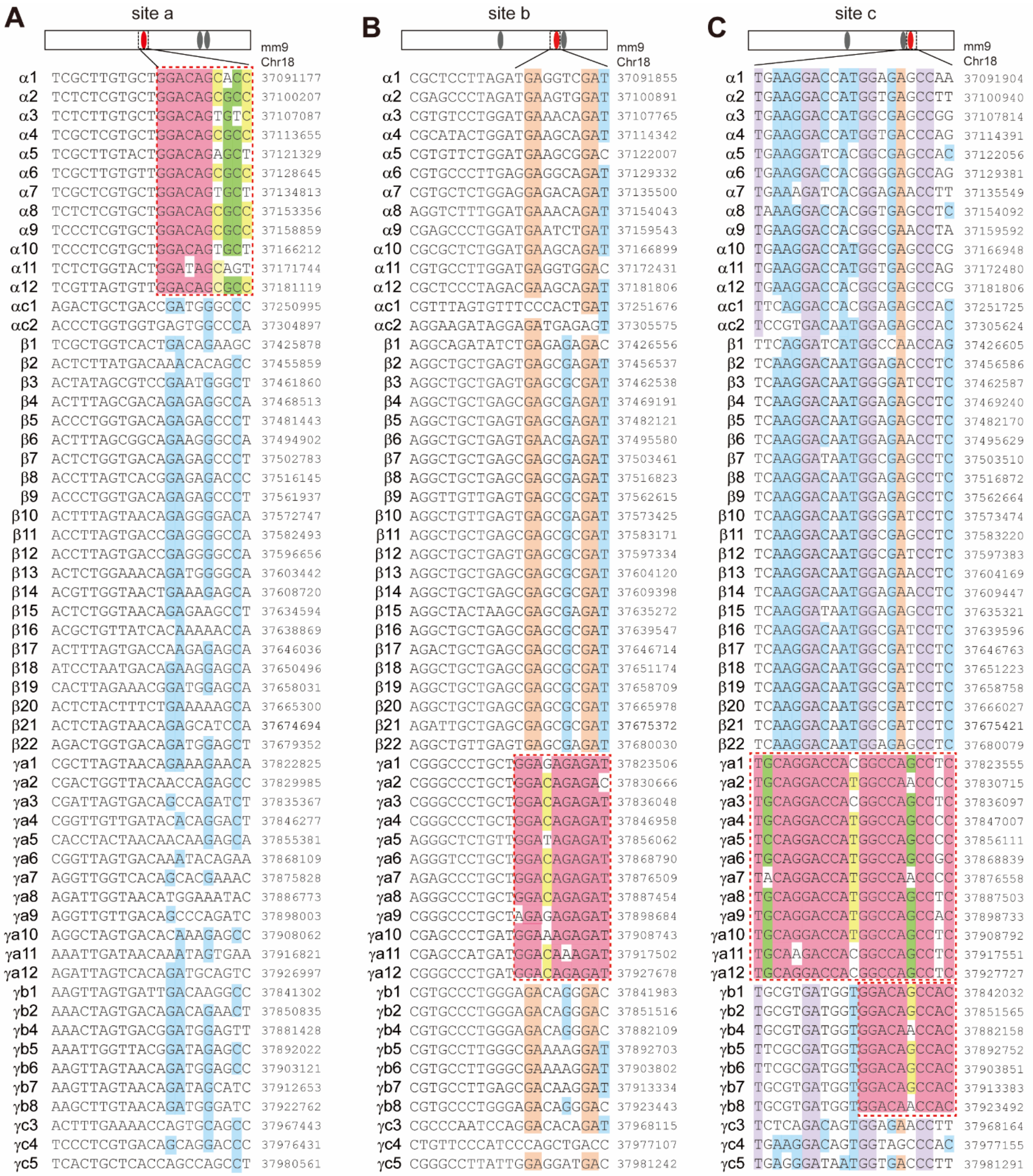

Supplementary Figure S9. Alignments and conserved genomic locations of the site ‘a’ (**A**), ‘b’ (**B**), and ‘c’ (**C**) of the clustered *Pcdh* variable exons in mice.
