# Supplementary material for: Mechanism of REST/NRSF regulation of clustered protocadherin α genes
Source: Nucleic Acids Res. 2021 Apr 13;49(8):4506–21. doi: 10.1093/nar/gkab248 (PMC8096226; doi:10.1093/nar/gkab248)
Supplement: gkab248_Supplemental_Files [file gkab248_supplemental_files.zip › Supplementary Table S1.pdf]

Supplementary Table S1. List of Primers Used

Name	5'-3' sequence
Primers used to generate plasmids for *in-vitro* expression of REST/NRSF proteins	
hREST(ZF1-8)-F	CGGAATTCACCATGGGTGCTCCAGATATTTACA
hREST(ZF2-8)-F	CGGAATTCACCATGTCCAAGGGCCCCATTCGC
hREST(ZF3-8)-F	CGGAATTCACCATGAATGAGCGAGTCTACAAG
hREST(ZF4-8)-F	CGGAATTCACCATGCCAAGGAAAGTATACACA
hREST(ZF5-8)-F	CGGAATTCACCATGGGAGAACGCCCATATAAAT
hREST(ZF1-8)-R	GCTCTAGATTACAGATCCTCTTCTGAGATGAGTTTTTG<br>TTCGG TAATATTATCAGGCAA
hREST(ZF1-7)-R	GCTCTAGATTACAGATCCTCTTCTGAGATGAGTTTTTG<br>TTCATTGAACTGCCGTGGGTT
hREST(ZF1-6)-R	GCTCTAGATTACAGATCCTCTTCTGAGATGAGTTTTTG<br>TTCAT<br>TAAGAGGTTTAGGCCC
hREST(ZF1-5)-R	GCTCTAGATTACAGATCCTCTTCTGAGATGAGTTTTTG<br>TTCTTTAAATGGCTTCTCACC
hREST(ZF1-4)-R	GCTCTAGATTACAGATCCTCTTCTGAGATGAGTTTTTG<br>TTCTTTATATGGGCGTTCTCC
hREST-ZF1m-F	ACCCTTTCGCCGTAAGCCACGCCAATATGA
hREST-ZF2m-R	TCATATTGGCGTGGCTTACGGCGAAAGGGT
hREST-ZF2m-F	CCCCATTCGCCGTGACCGCCGCGGCTACAA
hREST-ZF2m-R	TTGTAGCCGCGGCGGTCACGGCGAATGGGG
hREST-ZF3m-F	AGTCTACAAGCGTATCATTCGCACATACAC
hREST-ZF3m-R	GTGTATGTGCGAATGATACGCTTGTAGACT
hREST-ZF4m-F	AGTATACACACGCGGAAAACGCAACTATTT
hREST-ZF4m-R	AAATAGTTGCGTTTTCCGCGTGTGTATACT
hREST-ZF5m-F	CCCATATAAACGGGAACTTCGTCCTTACTC
hREST-ZF5m-R	GAGTAAGGACGAAGTTCCCGTTTATATGGG
hREST-ZF6m-F	GCCATTTAAACGCGATCAGCGCAGTTATGT
hREST-ZF6m-R	ACATAACTGCGCTGATCGCGTTTAAATGGC
hREST-ZF7m-F	ACCTCTTAATCGCCCACACCGTGATTACAA
hREST-ZF7m-R	TTGTAATCACGGTGTGGGCGATTAAGAGGT
hREST-ZF8m-F	GCAGTTCAATCGCCCTGTACGTGACTATGC
hREST-ZF8m-R	GCATAGTCACGTACAGGGCGATTGAACTGC

Primers used to clone the wild type REST/NRSF binding sites for EMSA	
EMSA-hPCDHα1-F	AGAACTGGCGGTCACTTCAT
EMSA-hPCDHα1-R	AGCTCACTGACTGCACCAATAG
EMSA-hPCDHα2-F	CCATCTCAGAGAACGCTTCC
EMSA-hPCDHα2-R	CTGCCAGTACCCAGCTGAAG
EMSA-hPCDHα3-F	GATCACTGCACAGTTCTACTCG
EMSA-hPCDHα3-R	GAGGAAGGCTAGGGCTAAAAG

EMSA-hPCDHα4-F	GCGTGTCCGACAAAGACATG
EMSA-hPCDHα4-R	CCTGTCCACTCTCCACAAGT
EMSA-hPCDHα5-F	GCCATAACCACCCTTTTCC
EMSA-hPCDHα5-R	TTGTAGCCCGAATCAGGGT
EMSA-hPCDHα6-F	TAATAGCCTTGTTGCAGCC
EMSA-hPCDHα6-R	GTGATGACGCCTTTGGAG
EMSA-hPCDHα7-F	CCAGGTACCGTCATCACATTG
EMSA-hPCDHα7-R	GTCAGCGTCAACTGCACGT
EMSA-hPCDHα8-F	TCAGAATCCAGAATGCCAGAC
EMSA-hPCDHα8-R	CTCATAGGCCGACACTCTC
EMSA-hPCDHα9-F	TTAGTGTGATCGACCTAGACG
EMSA-hPCDHα9-R	ACGCCCGCGACGATGACTTT
EMSA-hPCDHα10-F	CCTAATCAGCGTTTCTGACCAT
EMSA-hPCDHα10-R	CACATCTACCATTGGGCATG
EMSA-hPCDHα11-F	TCTCCTGAAGTCGCCGTG
EMSA-hPCDHα11-R	GACCGCGGTACTAGCTTGTT
EMSA-hPCDHα12-F	CAATGTCCCTGAAGTAATGGTTAC
EMSA-hPCDHα12-R	CGTAGGACAGCCAAGCGTTAT
EMSA-hPCDHα13-F	TAACGCCCCAGAGGTTACC
EMSA-hPCDHα13-R	CCCTCGACGAAGCCTGT
EMSA-hPCDHβ4-F	ATGCTCCTGAGACGGTAGTCT
EMSA-hPCDHβ4-R	TCAGGAACTTGAACTCACCAG
EMSA-hPCDHβ7-F	ATCGACCCGAGCTGCTCC
EMSA-hPCDHβ7-R	GCCTGTGCTCTGGGGTAGC
EMSA-hPCDHγa1-F	CTCTTATCAGTGTGCATGACCAG
EMSA-hPCDHγa1-R	GTAAAGACTGGGGTGCTGATAG
EMSA-hPCDHγa2-F	GTTGCGAGACTTGCAAGTGT
EMSA-hPCDHγa2-R	TTCGAGTAGAGATTGAGGCG
EMSA-hPCDHγa3-F	CAACGCCCGCATCACTTATG
EMSA-hPCDHγa3-R	TGCGAGCCGGGCGTACT
EMSA-hPCDHγa4-F	CACTTTTCAACGTGCATGACAGT
EMSA-hPCDHγa4-R	TGAGGCCCGAGTCGTCTG
EMSA-hPCDHγa5-F	GCTGTTTAGCGTACATGATGGT
EMSA-hPCDHγa5-R	GGTCAATGGGGGTCTTGATAC
EMSA-hPCDHγa6-F	GGAATTCAACATGGCGCCTCCGCAGA
EMSA-hPCDHγa6-R	GGGAAGCTTTTACTTCTTCTCCTTCTTGCCCG
EMSA-hPCDHγa7-F	GTGACTGCACATGACAGCG
EMSA-hPCDHγa7-R	CACGGTGAGTGTGACGGT
EMSA-hPCDHγa8-F	CTGTGACCGAGGACACGCT
EMSA-hPCDHγa8-R	GTGTAAGGCTCGAATCGTTCG
EMSA-hPCDHγa9-F	GAGATTTGCAAATGCAGGTG
EMSA-hPCDHγa9-R	TACTGTGAGCGTGACAGTGG
EMSA-hPCDHγa10-F	CTTCTCAGTGACAGCGCTG
EMSA-hPCDHγa10-R	GGAACCAATGGGGTATCCTC

EMSA-hPCDHγa11-F	GATTGCTCTTCTAAATGTGCAAG
EMSA-hPCDHγa11-R	CACCACCAAGTACAGCGAG
EMSA-hPCDHγa12-F	CACCATCCAAGGGGCAAG
EMSA-hPCDHγa12-R	AATACCGAATCACCTGACAGC
EMSA-hPCDHγb1-F	GGGACCCAACGGCAGAG
EMSA-hPCDHγb1-R	AGCAGCCCTCAGTGTCGAG
EMSA-hPCDHγb2-F	CCTGACTTGGGCCCCAGT
EMSA-hPCDHγb2-R	GGGGTCAGAGGGCTCCC
EMSA-hPCDHγb3-F	CCTGGCTTCTGAATCCCAAC
EMSA-hPCDHγb3-R	GAAACTGTAGCTCCGCCTGAG
EMSA-hPCDHγb4-F	CTATTTCACAAGTCAGGGCTTC
EMSA-hPCDHγb4-R	AACCACGGATTCGGATTTAAC
EMSA-hPCDHγb5-F	GACCTAGAGCCTCTGGCAC
EMSA-hPCDHγb5-R	GATGGGAAGTCGACTCGC
EMSA-hPCDHγb6-F	GGGCTCAATGGCCACAT
EMSA-hPCDHγb6-R	GATGGAAGCAGTCCCAAGTAG
EMSA-hPCDHγb7-F	ACCTGGAGTCACGAACGC
EMSA-hPCDHγb7-R	GAGTCAGAGGGTGTGGGATG
EMSA-hPCDHαc1pro-F	TAAGATCTGGGCAGCCTCAG
EMSA-hPCDHαc1pro-R	GTCCACGTTCCACCAACAC
EMSA-hPCDHαc1-F	CCTCCCAGAAGTGCAACAG
EMSA-hPCDHαc1-R	CAGAAAGCTGCCCTCCTG
EMSA-hPCDHαc2pro-F	GGGAGCTGATAGCCAGACTTC
EMSA-hPCDHαc2pro-R	CGCTCACGCTCTAGAAAGC
EMSA-hPCDHγc3-F	GAGATTAGCGAGGCCGTG
EMSA-hPCDHγc3-R	CGGACACTTGAACACGCAC
EMSA-hPCDHγc4-F	GTGAACCAAAGACACTTCCGT
EMSA-hPCDHγc4-R	ACCAGGCGGTAGTCCGAT
EMSA-hPCDHγc5-F	CTATTTTTCCCTGAGCTTGATG
EMSA-hPCDHγc5-R	TCCCACACGTAGAACTGAGG
EMSA-hPCDHHS7-F	GGATTAACTCTTGGCAGTCCTG
EMSA-hPCDHHS7-R	GCAGAGCTGACACAATAGCAC
EMSA-hPCDHSE#1/2-F	CCGTATCACTGATACCTTGGC
EMSA-hPCDHSE#1/2-R	TGAGTGATGTATGCATGGGG
EMSA-hPCDHSE#3/4/5-F	GCTGCAACAGGACAAGATCC
EMSA-hPCDHSE#3/4/5-R	CCAAAGGTCAGGCATCAGG
EMSA-hPCDHSE#6/7/8-F	GGTGACCCCTATATTCCCAGTGC
EMSA-hPCDHSE#6/7/8-R	TTCTCTGGCAGCCCCGCT
EMSA-hPCDHSE#9/10-F	TGCTTGTGAAGTCCAGAGTAAGG
EMSA-hPCDHSE#9/10-R	CCGGGTCAAGCAAATGAG
EMSA-hPCDHSE#11/12/13-F	TGATGGACATAGGGGTGTTCAT
EMSA-hPCDHSE#11/12/13-R	TTTATCTTGGGCAGAGCAAGTAG
EMSA-hPCDHSE#14-F	CTGTATCTCACCCACGCTAACC
EMSA-hPCDHSE#14-R	GCAAGATGGGATGGAACCAT

EMSA-hHS5-1-F	TACCGCTCGAGAATACTACTAGGCACTTGTTTGGATC
EMSA-hHS5-1-R	GAGCGAGCTCTGGCTAACAACATAGTGCTTCCT
EMSA-CELSR3-F	TCGCCGAGGTTACTTTCCTG
EMSA-CELSR3-R	TTTTTGATTCGGCACCACGG
EMSA-MAPK8IP-F	ACATCCTCTCCTTTCTCTGCC
EMSA-MAPK8IP-R	GCAGCCAATGCGGATCAGT
EMSA-synapsin1-F	CCTAGATTGGCGTGTGTTCTG
EMSA-synapsin1-R	AGGTAGTTCATGGCTGCGAC

Primers used to mutate REST/NRSF binding sites for EMSA

EMSA-hPCDHα8-M1R	TAGGCCGACACTCTCTCGCGGTCCAGGGCGCTGGAAAGCAC
EMSA-hPCDHα8-M2R	CTCATAGGCCGACACTCTCTCGCGGTCCAGGGCGAGTTCC
EMSA-hPCDHα8-M3R	TAGGCCGACACTCTCTCGCGGTCCAGGTATCTGTCCAGCAC
EMSA-hPCDHα8-M11R	TAGGCCGACACTCTCTCGCGGTCCCTTGCGCTGTCC
EMSA-hPCDHα8-M12R	TAGGCCGACACTCTCTCGCGGGAAAGGGCGCTGTCC
EMSA-hHS5-1-M1R	TAAAGGAAATCTCTCTGGCCACGCCCAGAGTTCAGCACCATGGCCACTTCCAGCG
EMSA-hHS5-1-M2R	TAAAGGAAATCTCTCTGGCCACGCCCAGAGTTCAGCACCATGGCCACGGAACTCGACA
EMSA-hHS5-1-M3R	TAAAGGAAATCTCTCTGGCCACGCCCAGAGTTCAGCACCATGGCCACGGACAGATCCAC
EMSA-hHS5-1-M4R	TAAAGGAAATCTCTCTGGCCACGCCCAGAGTTCAGCCAAATGGC
EMSA-hHS5-1-M5R	TAAAGGAAATCTCTCTGGCCACGCCCAGAGTTCCTAACCAT
EMSA-hHS5-1-M6R	TAAAGGAAATCTCTCTGGCCACGCCCAGAGGGAAGCAC
EMSA-hHS5-1-M7R	TAAAGGAAATCTCTCTGGCCACGCCCAGAGTTCAGCACACGGGCCA
EMSA-hHS5-1-M8R	TAAAGGAAATCTCTCTGGCCACGCCCAGAGTTCAGACACATGG
EMSA-hHS5-1-M9R	TAAAGGAAATCTCTCTGGCCACGCCCAGAGTTACTCACCA
EMSA-hHS5-1-M10R	TAAAGGAAATCTCTCTGGCCACGCCCAGATGGCAGCA

Primers used to amplify probes for EMSA

hPCDHα1/2/4/5/6/8/11a-NRSE-F†	CACCTTCAAGAATTACTACTCG
hPCDHα1/5a-NRSE-R	CTCATAGACCGACAGGCT
hPCDHα2/4a-NRSE-R	CTCATAGGCTGACACGCT
hPCDHα3/6/12a-NRSE-R	CTCATAGGCCGACACGCT
hPCDHα8a-NRSE-R	CTCATAGGCCGACACTCTC
hPCDHα11a-NRSE-R	TTCATAGGCCCACACGTT

hPCDHα3/10a-NRSE-F†	CACCTACAAGAATTACTA
hPCDHα10a-NRSE-R	CTCATAGGCCGACACCCT
hPCDHα7a-NRSE-F†	CACCTTCAAGAATTACTATTC
hPCDHα7a-NRSE-R	CTCATAGGCGGACACACT
hPCDHα9/12a-NRSE-F†	CACCTACAAGAATTACTACTCG
hPCDHα9a-NRSE-R	CTCGTAGGCGGACACACT
hPCDHα13a-NRSE-F†	CACCTACAAGAACTACTACTC
hPCDHα13a-NRSE-R	TTCATAGGCTGATACGCT
hPCDHβ7aCtr-NRSE-F†	CTTCTATACTCTGGTAACAG
hPCDHβ7aCtr-NRSE-R	GGTGATGGTGATGTTGTACT
hPCDHγa4aCtr-NRSE-F†	TTATTATCGGTTGTTGACAC
hPCDHγa4aCtr-NRSE-R	AGTTACAGTGATGTTATATTC
hPCDHγb3aCtr-NRSE-F†	CACATACAGGTTGGTGAC
hPCDHγb3aCtr-NRSE-R	TGTGATCGTCACATTGTATTC
hPCDHγa1/2/3/6/7/8/10/12b-NRSE-F†	GGGCGAGGTGCGCACGGC
hPCDHγa1b-NRSE-R	GACGGCCACCACGAGACT
hPCDHγa2b-NRSE-R	GATGGCCACCACGAGGCT
hPCDHγa3/5/7/8/11b-NRSE-R	GACGGCCACCACGAGGCT
hPCDHγa6b-NRSE-R	GACGGCCACCACTAGGCTCTGC
hPCDHγa10/12b-NRSE-R	GACGGCCACTACGAGGCT
hPCDHγa4b-NRSE-F†	AGGCGAGGTGCGCACCGC
hPCDHγa4b-NRSE-R	GACGACCACTACAAGCCT
hPCDHγa5b-NRSE-F†	GGGCGAGGTGCGCACAGC
hPCDHγa9b-NRSE-F†	AGGTGAAGTGCGCACAGC
hPCDHγa9b-NRSE-R	TACAGCCACCACAAGGCT
hPCDHγa11b-NRSE-F†	GGGCGAGGTGCGTACAGC
hPCDHβ4bCtr-NRSE-F†	TGGCGAGGTGCGCACCGC
hPCDHβ4bCtr-NRSE-R	GACAAGCACCACGAGCCT
hPCDHγb3bCtr-NRSE-F†	GGGTGAGGTGCGCACGGC
hPCDHγb3bCtr-NRSE-R	CACGGCGACCAGAAGGCG
hPCDHαc1bCtr-NRSE-F†	AGGTGAGCTCCGTACTGC
hPCDHαc1bCtr-NRSE-R	AACCACTACCACCACCCT
hPCDHγa1c-NRSE-F†	ACGCGCTCAAGCAGAGGC
hPCDHγa1/2/3/6/10/12c-NRSE-R	CCACGGTGAGCGTGACAGT
hPCDHγa2/3/5/6/11/12c-NRSE-F†	ACGCGCTCAAGCAGAGCC
hPCDHγa4c-NRSE-F†	ACGCGCTCAAGCAGAGGC
hPCDHγa4c-NRSE-R	CCACAGTGAGTGTGACGG
hPCDHγa5c-NRSE-R	CAACGGTGACCGTGAAGG
hPCDHγa7c-NRSE-F†	ATGCCCTCAAGCAGAGCC
hPCDHγa7c-NRSE-R	CCACGGTGAGTGTGACGGT
hPCDHγa8c-NRSE-F†	ATGCGCTCAAGCAGAGCC
hPCDHγa8c-NRSE-R	CTACGGTGAGCGTGACAG
hPCDHγa9c-NRSE-F†	ATGCGCTCAAACAGAGCC
hPCDHγa9c-NRSE-R	CTACTGTGAGCGTGACAG

hPCDHγa10c-NRSE-F†	ACGCGCTCAAGCAAAGCC
hPCDHγa11c-NRSE-R	CCACGGTGAGCGTGACGG
hPCDHγb1/2/5c-NRSE-F†	ACGCGGCCCGCCAGCGCC
hPCDHγb1c-NRSE-R	TTAGGTGCAGCGTGGCGG
hPCDHγb2c-NRSE-R	TTAGGTGCAGCGTGGCCG
hPCDHγb3c-NRSE-F†	AGGCCGCCCGCCAGCGCC
hPCDHγb3c-NRSE-R	TTAGGTGCAGCATGACGG
hPCDHγb4c-NRSE-F†	ACGCCGTCCGCCAGCGCC
hPCDHγb4c-NRSE-R	CCAGGTGCAACGTGGCAG
hPCDHγb5c-NRSE-R	CCAAGTGCAGCGTGGCGG
hPCDHγb6c-NRSE-F†	ACGCAGCCCGCCAGCGCC
hPCDHγb6c-NRSE-R	CCAGATGAAGCGTGGCGG
hPCDHγb7c-NRSE-F†	ACTCGGTCCGCCAGCGCC
hPCDHγb7c-NRSE-R	CCAGGTGCAGCGTGGCAG
hPCDHα4cCtr-NRSE-F†	ACGCTCCGCGCCACCGCC
hPCDHα4cCtr-NRSE-R	ACACCAGCACAGTGGCCG
hPCDHβ4cCtr-NRSE-F†	ACGCAGCCAAGCACAGGC
hPCDHβ4cCtr-NRSE-R	GCACGTGCAGCGTGGCGG
hPCDHαc1pro-NRSE-F†	TGGTCGAGACCCCAGCCC
hPCDHαc1pro-NRSE-R	CAGAGACGAGGCCGCCCG
hPCDHαc2pro-NRSE-F†	GATGGGGCTGGAGAGGCT
hPCDHαc2pro-NRSE-R	GGTAGGAGGGCTCAGCAAG
hPCDHγc3-NRSE-F†	TGAGCCGAAATGAATACT
hPCDHγc3-NRSE-R	GGCGCGCTCCAACACCAG
hPCDHγc4-NRSE-F†	GCGGCAGCAGCTGGACTT
hPCDHγc4-NRSE-R	CATCTGCATCCTGAGCCT
hPCDHγc5-NRSE-F†	ATCAGCAGCATCTGGGGCAC
hPCDHγc5-NRSE-R	CACAGTATTGGTGCCCAC
hHS7#1-NRSE-F†	GCTCTGAGGGCAACTAAAG
hHS7#1-NRSE-R	AAACCTCTGATTCGGCAC
hHS7#2-NRSE-F†	CATTTCTCTCTTTTGTCTCAG
hHS7#2-NRSE-R	CAGAATCTGCCTGTTTACC
hSE#1-NRSE-F†	TTCTAGGATGTGGGTGG
hSE#1-NRSE-R	GCTGACATACCTGAGTCCT
hSE#2-NRSE-F†	AGGCACTATCCCAAACAAGG
hSE#2-NRSE-R	TCTGCCCTCTAGCCTATTGT
hSE#3-NRSE-F†	ATTTCCTGACAGGTAGAGGG
hSE#3-NRSE-R	GATGTCATCACGTTAGAGAG
hSE#4-NRSE-F†	CTAAAATAAAAGGGATCCAT
hSE#4-NRSE-R	ATTTTATTTCTAGCAGTGTGC
hSE#5-NRSE-F†	CTCCAATAGTAAGTCTTCTGT
hSE#5-NRSE-R	GGGGCAAAACTTTAAAACT
hSE#6-NRSE-F†	TTCCCAGTGCTCAGCGT
hSE#6-NRSE-R	CTTATATCCCCAGCAGTTAATG

hSE#7-NRSE-F†	CTCCCCTTGGGAAACAG
hSE#7-NRSE-R	CATATTCAACCCCCTGG
hSE#8-NRSE-F†	CCCCAGTGCCTCCTTGTGC
hSE#9-NRSE-F†	ATCAACTTTTGTTCATACACAC
hSE#9-NRSE-R	AAGCAGATATTTGGAACAAAG
hSE#10-NRSE-F†	GTGAAATCCCAAAAAGTCCATAG
hSE#11-NRSE-F†	CGGTTTCAAAAATAACAT
hSE#11-NRSE-R	AATCAAATGCCCCAAG
hSE#12-NRSE-F†	TCCTGCGTCACAAATAC
hSE#12-NRSE-R	CATAAAACAGACTCCCTATCT
hSE#13-NRSE-F†	CAGGCAGCTCCACCTAACCT
hSE#14-NRSE-F†	ACTGCTGTTTTTTTCCCTTCT
hSE#14-NRSE-R	CCCATCTCCACCCCTTC
hHS5-1- NRSE-F†	GCAGCGAGTCATGGGACC
hHS5-1- NRSE-R	TAAAGGAAATCTCTCTGGCC
Celsr3-I-NRSE-F†	TGTCTTCCAGGGGCCTCG
Celsr3-I-NRSE-R	AACCTGATGCAGGAGCTGTC
Celsr3-II-NRSE-F†	CTGCATCAGGTTCAGCACC
Celsr3-II-NRSE-R	AAGAGACCCCGGGAGCG
Celsr3-tandem-NRSE-F†	CTGGATTCAGCACCACGCAC
Celsr3-tandem-NRSE-R	GCTCGGGAGCTGTCCGAG
MAPK8IP-NRSE-F†	AACCAAGCCCAGGGCCGC
MAPK8IP-NRSE-R	CCCCACCCCGCCAGCCCC
synapsin1-NRSE-F†	GATGCGGCGAGGCGCGTG
synapsin1-NRSE-R	CGGTGGCGCGCGCCGCCA
hPCDHγa6-tandem-NRSE-F†	GCGCCCTGCTGGACAGAG
hPCDHγa6-tandem-NRSE-R	GCGGAGAGAGGGGGCTGG
hPCDHγa7-tandem-NRSE-F†	GGGCCCTGCTGGACAGAG
hPCDHγa7-tandem-NRSE-R	GCTGACAGAGGAGGCTGAC

Primers used to generate plasmids for shRNA knockdown

REST-shRNA1-F	CCGGGCAAACACCTCAATCGCCATTCTCGAGAATGGCG ATTGAGGTGTTTGCTTTTTG
REST-shRNA1-R	AATTCAAAAAGCAAACACCTCAATCGCCATTCTCGAGA ATGGCGATTGAGGTGTTTGC
REST-shRNA2-F	CCGGCATGCAAGACAGGTTCACAATCTCGAGATTGTGA ACCTGTCTTGCATGTTTTTG
REST-shRNA2-R	AATTCAAAAACATGCAAGACAGGTTCACAATCTCGAGA TTGTGAACCTGTCTTGCATG
REST-shRNA3-F	CCGGGGAGCAAGTCCTTATTGAAGTCTCGAGACTTCAA TAAGGACTTGCTCCTTTTTG
REST-shRNA3-R	AATTCAAAAAGGAGCAAGTCCTTATTGAAGTCTCGAGA CTTCAATAAGGACTTGCTCC
GFP-shRNA-F	CCGGGTCGAGCTGGACGGCGACGTACTCGAGTACGTCG

	CCGTCCAGCTCGACTTTTTG
GFP-shRNA-R	AATTCAAAAAGTCGAGCTGGACGGCGACGTACTCGAGTACGTCGCCGTCCAGCTCGAC

Primers used for construction of sgRNA expressing plasmids and for genotyping

hHS51-NRSE-sgRNA1-F	ACCGACAGCGACACCGCCCAGTT
hHS51-NRSE-sgRNA1-R	AAACAACTGGGCGGTGTCGCTGT
hHS51-NRSE-sgRNA2-F	ACCGTGGCCATGGTGCTGAACTC
hHS51-NRSE-sgRNA2-R	AAACGAGTTCAGCACCATGGCCA
hHS51-NRSE-PCR-F	CTTGGAACCAGTTGGGATTG
hHS51-NRSE-PCR-R	TTATCAATAGCATTTTCCTCATCTG
mHS51-NRSE-sgRNA1-F	TAATACGACTCACTATAGGGCGAGTCATGGGACTCAACT
mHS51-NRSE-sgRNA2-F	TAATACGACTCACTATAGGATCTGGGGTGCTGAATCCT
Mouse-sgRNA-R	AAAAGCACCGACTCGGTGCC
mHS51-NRSE-PCR-F	GTTCCTTTGTCAGGTGAAAATCT
mHS51-NRSE-PCR-R	GGGTGGTAGTGAGGGATTATTCTAG

Primers used for QHR-4C

4C-adaptorF	GACGTGTGCTCTTCCGATCTGNNNNNN
4C-adaptorR‡	CAGATCGGAAGAGCACACGTC
4C-hHS51-bioprimer†	TGCTTTCTCATTCCCCGTTG
4C-hHS51-DpnII-F1	AATGATACGGCGACCACCGAGATCTACACTCTTTCCCTACACGACGCTCTTCCGATCTCAGTTTTGGCGGCGACAAATTCG
4C-hHS51-DpnII-F2	AATGATACGGCGACCACCGAGATCTACACTCTTTCCCTACACGACGCTCTTCCGATCTCACGTTTTGGCGGCGACAAATTCG
4C-hα12-bioprimer†	CCGCACCCACATTCCAATCA
4C-hα12-DpnII-F1	AATGATACGGCGACCACCGAGATCTACACTCTTTCCCTACACGACGCTCTTCCGATCTTCCAATCATTCACGGAATAGGATC
4C-hα12-DpnII-F2	AATGATACGGCGACCACCGAGATCTACACTCTTTCCCTACACGACGCTCTTCCGATCTGTCCAATCATTCACGGAATAGGATC
4C-mHS51-bioprimer†	GCTTTGTTACTCTAGGAACAG
4C-mHS51-DpnII-F1	AATGATACGGCGACCACCGAGATCTACACTCTTTCCCTACACGACGCTCTTCCGATCTGGAGGTTAAAGCAAAGACTAAGATC
4C-mHS51-DpnII-F2	AATGATACGGCGACCACCGAGATCTACACTCTTTCCCTACACGACGCTCTTCCGATCTGAGGAGGTTAAAGCAAAGACTAAGATC
4C- mα9-bioprimer†	GATATAGTTCGCTGTTTCTCAGGG
4C-mα9-DpnII-F1	AATGATACGGCGACCACCGAGATCTACACTCTTTCCCT

	ACACGACGCTCTTCCGATCTTAGGAAGTAGCTACGTTC GGAG
P7-index-R1	CAAGCAGAAGACGGCATACGAGATCTGCTAGTGACTGG AGTTCAGACGTGTGCTCTTCCGATCT
P7-index-R2	CAAGCAGAAGACGGCATACGAGATGTCTAGGTGACTGG AGTTCAGACGTGTGCTCTTCCGATCT
P7-index-R3	CAAGCAGAAGACGGCATACGAGATTCGTACGTGACTGG AGTTCAGACGTGTGCTCTTCCGATCT
P7-index-R4	CAAGCAGAAGACGGCATACGAGATAGTACGGTGACTGG AGTTCAGACGTGTGCTCTTCCGATCT
P7-index-R5	CAAGCAGAAGACGGCATACGAGATGACCTAGTGACTGG AGTTCAGACGTGTGCTCTTCCGATCT
P7-index-R6	CAAGCAGAAGACGGCATACGAGATGAGTTCGTGACTGG AGTTCAGACGTGTGCTCTTCCGATCT
P7-index-R7	CAAGCAGAAGACGGCATACGAGATGAAGCTGTGACTGG AGTTCAGACGTGTGCTCTTCCGATCT
P7-index-R8	CAAGCAGAAGACGGCATACGAGATCTTGCAGTGACTGG AGTTCAGACGTGTGCTCTTCCGATCT

Primers used for bisulfite sequencing

mα9_BSP_F	TAGAAGTAAATTAAAGATGTGAATTAAAGA
mα9_BSP_R	ACCTCAAACATTTAAAAATCACCAC

† 5' biotin labeled

‡ phosphorylated
